# Lipidomics Reveals Serum Specific Lipid Alterations in Diabetic Nephropathy

**DOI:** 10.3389/fendo.2021.781417

**Published:** 2021-12-09

**Authors:** Tingting Xu, Xiaoyan Xu, Lu Zhang, Ke Zhang, Qiong Wei, Lin Zhu, Ying Yu, Liangxiang Xiao, Lili Lin, Wenjuan Qian, Jue Wang, Mengying Ke, Xiaofei An, Shijia Liu

**Affiliations:** ^1^ Affiliated Hospital of Nanjing University of Chinese Medicine, Nanjing, China; ^2^ Core Facility Center, CAS Center for Excellence in Molecular Plant Sciences, Chinese Academy of Sciences, Shanghai, China; ^3^ Renal Division, The 3^rd^ Xiangya Hospital-Central South University, Changsha, China; ^4^ Department of Endocrinology, Zhongda Hospital Southeast University, Nanjing, China; ^5^ Division of Nephrology, Tongji Hospital, Tongji University School of Medicine, Shanghai, China; ^6^ Division of Nephrology, Zhongshan Hospital, Xiamen University School of Medicine, Xiamen, China; ^7^ College of Pharmacy, Jiangsu Collaborative Innovation Center of Chinese Medicinal Resources Industrialization, Nanjing University of Chinese Medicine, Nanjing, China; ^8^ Jiangsu Key Laboratory of Traditional Chinese Medicine (TCM) Evaluation and Translational Research, China Pharmaceutical University, Nanjing, China

**Keywords:** Lipidomics, LPE(16:0), PE(16:0/20:2), TAG54:2-FA18:1, diabetic nephropathy

## Abstract

In diabetes mellitus (DM), disorders of glucose and lipid metabolism are significant causes of the onset and progression of diabetic nephropathy (DN). However, the exact roles of specific lipid molecules in the pathogenesis of DN remain unclear. This study recruited 577 participants, including healthy controls (HCs), type-2 DM (2-DM) patients, and DN patients, from the clinic. Serum samples were collected under fasting conditions. Liquid chromatography-mass spectrometry-based lipidomics methods were used to explore the lipid changes in the serum and identify potential lipid biomarkers for the diagnosis of DN. Lipidomics revealed that the combination of lysophosphatidylethanolamine (LPE) (16:0) and triacylglycerol (TAG) 54:2-FA18:1 was a biomarker panel for predicting DN. The receiver operating characteristic analysis showed that the panel had a sensitivity of 89.1% and 73.4% with a specificity of 88.1% and 76.7% for discriminating patients with DN from HCs and 2-DM patients. Then, we divided the DN patients in the validation cohort into microalbuminuria (diabetic nephropathy at an early stage, DNE) and macroalbuminuria (diabetic nephropathy at an advanced stage, DNA) groups and found that LPE(16:0), phosphatidylethanolamine (PE) (16:0/20:2), and TAG54:2-FA18:1 were tightly associated with the stages of DN. The sensitivity of the biomarker panel to distinguish between patients with DNE and 2-DM, DNA, and DNE patients was 65.6% and 85.9%, and the specificity was 76.7% and 75.0%, respectively. Our experiment showed that the combination of LPE(16:0), PE(16:0/20:2), and TAG54:2-FA18:1 exhibits excellent performance in the diagnosis of DN.

## Introduction

As a significant microvascular complication of diabetes mellitus (DM), both type 1 and type 2, diabetic nephropathy (DN) has become the leading cause of chronic kidney disease (CKD) (1, 2). DN is characterized by dysfunction of the glomerular filtration barrier and decreased kidney function, which could be directly reflected by the persistent elevation of albumin in the urine and a progressive decrease in estimated glomerular filtration rate (eGFR), respectively (3). By 2019, there were approximately 463 million DM patients worldwide, among which type-2 DM (2-DM) accounted for more than 90% (4). It is estimated that 25–40% of diagnosed DM patients will eventually develop DN (5). Meanwhile, DN is an independent risk factor for increased mortality from cardiovascular causes, such as myocardial infarction, sudden cardiac death, stroke, and other fatal complications of diabetic cardiomyopathy (6).

In the clinic, microalbuminuria is considered the earliest evidence of the onset of DN. It has been reported that microalbuminuria progresses to macroalbuminuria in 50% of diagnosed DN patients without effective intervention and eventually develops into end-stage renal disease (ESRD) (7, 8). Undoubtedly, albuminuria is a significant sign of DN. However, the development of kidney impairment in DM patients is not synchronized with the increase in albuminuria (9). According to the national health and nutrition examination survey (NHANES), the number of DN patients with an eGFR of < 60 ml/min/1.73 m^2^ but without albuminuria has increased over the past 30 years (10). In addition, these patients’ annual mortality rate increased from 3.5% to 5.1% during this period (11). At present, the urine albumin creatine ratio (UACR) and eGFR are broadly applied parameters for diagnosing the initiation and progression of DN in the clinic. Nevertheless, in most DN patients during the early stages, their urinary albumin or eGFR level is normal. It has also been reported that the levels of microalbuminuria in some DN patients who received or did not receive intervention treatment returned to baseline rather than progressing to macroalbuminuria (12–14). Therefore, it is urgently necessary to develop more accurate diagnostic markers for DN in the clinical setting.

Lipid molecules are ubiquitous in all organisms and they make up essential components of cell membranes, lipid particles, and nerve myelin sheaths (15). Their functions include serving as cell barriers, membrane matrix, signal transduction, and energy storage (16). In 2005, the LIPID MAPS consortium classified lipids into eight categories based on their chemical and biochemical characteristics, which contains tens to hundreds of thousands of molecular species (17). Lipids are highly complex and dynamic, changing with physiological, pathological, and environmental conditions (18). In particular, lipid metabolites can serve as signaling molecules to activate multiple signaling pathways, thereby regulating cell growth, proliferation, and differentiation (19–21). Lipid disorders are associated with many diseases, such as Alzheimer’s disease, metabolic disorders, cancer, and kidney disease (22–24). Lipidomics is the systematic analysis of lipids in the entire organism. It reveals the mechanism of lipids in various life activities (25). A previous urinary exosomal lipidomics study on DM and DN revealed that diacylglycerol (DAG), triacylglycerol (TAG), ganglioside GM3, and lysophosphatidylcholine (LPC) were significantly upregulated in DN patients (26).

In this study, we aimed to analyze the serum lipid characteristics in HCs, 2-DM patients, and DN patients by liquid chromatography-mass spectrometry metabolomics (LC–MS). The aim was to evaluate the effects of lipid metabolism on DN development, to understand the mechanisms of metabolic disorders in DN, and to identify potential lipid biomarkers for DN.

## Materials And Methods

### Ethics Compliance Statement

All procedures were approved by the Institutional Review Board and the Ethics Committee of the First Affiliated Hospital of Nanjing University of Traditional Chinese Medicine (2019NL-109–02), registered in the Chinese Clinical Trial Registry (ChiCTR2000028949), and followed the Declaration of Helsinki. After reviewing the study’s written plan, all participants signed written informed consent before inclusion.

### Study Population

A total of 577 participants, including healthy controls (HCs), patients with type 2 diabetes mellitus (2-DM), and diabetic nephropathy (DN), including microalbuminuria (diabetic nephropathy at an early stage, DNE) and macroalbuminuria (diabetic nephropathy at an advanced stage, DNA), from the Affiliated Hospital of Nanjing University of Chinese Medicine, were enrolled. All of the participants were Asian and met the diagnostic criteria of 2-DM, and the patients with DNE and DNA met the diagnostic criteria of DN. All serum samples were collected under fasting conditions, and the classification of DN was made according to UACR. In this study, we defined patients with UACR<30 mg/g as having 2-DM and 30≤UACR mg/g as having DN (30≤UACR ≤ 300 mg/g as having DNE, and UACR>300 mg/g as having DNA). The analytical sample included 169 healthy subjects, 170 participants with 2-DM, 238 participants with DN, including 64 participants with DNE, and 64 participants with DNA in the validation cohort. The clinical information of all participants, including all examination indicators, is recorded in **Table 1**. Serum samples were collected and stored at -80°C until further analysis.

**Table 1:** Characterization of the study participants.

Covariate	Discovery Set (n = 330)			Validation Set (n = 247)			
	HCs	2-DM	DN	HCs	2-DM	DNE	DNA
Number	110	110	110	59	60	64	64
Male/Female	52/58	72/38	67/43	38/21	39/21	35/29	42/22
Age (years)	31.20 ± 8.4	53.75 ± 10.9	57.88 ± 10.2	34.47 ± 9.2	56.65 ± 10.9	53.38 ± 13.0	65.55 ± 12.2
BMI (kg/m^2^)	21.71 ± 2.9	24.51 ± 5.3	25.61 ± 5.1	22.14 ± 2.9	25.18 ± 2.8	31.84 ± 44.9	25.94 ± 4.1
HbA1c (%)	—	6.2 ± 4.1	6.2 ± 4.0	—	8.8 ± 2.0	9.2 ± 2.0	7.6 ± 1.5
eGFR (ml/min/1.73m^2^)	—	99.52 ± 14.0	74.75 ± 37.8	—	100.76 ± 14.0	99.89 ± 22.9	32.55 ± 25.7
ALB (g/L)	44.54 ± 2.4	38.88 ± 2.9	35.77 ± 6.0	42.03 ± 5.5	39.54 ± 4.3	38.90 ± 3.4	30.15 ± 4.7
BUN (mmol/L)	5 ± 1	7 ± 2	10 ± 6	5 ± 1	6 ± 2	7 ± 3	16 ± 7
Scr (μmol/L)	67 ± 12	68 ± 15	136 ± 152	68 ± 13	63 ± 12	67 ± 22	262 ± 172
Glu (mmol/L)	5 ± 0	8 ± 3	8 ± 3	5 ± 0	8 ± 3	10 ± 4	7 ± 4
Uric acid (μmol/L)	287 ± 69	308 ± 93	352 ± 141	289 ± 69	290 ± 97	326 ± 106	453 ± 121
Total cholesterol (mmol/L)	4 ± 1	4 ± 1	5 ± 2	5 ± 0	4 ± 1	5 ± 1	5 ± 2
Triglycerides (mmol/L)	1 ± 0	2 ± 4	2 ± 2	1 ± 0	2 ± 2	3 ± 3	2 ± 1
HDL cholesterol (mmol/L)	2 ± 0	1 ± 0	1 ± 0	2 ± 0	1 ± 0	1 ± 0	1 ± 0
LDL cholesterol (mmol/L)	2 ± 1	3 ± 1	3 ± 1	3 ± 0	3 ± 1	3 ± 1	3 ± 1
ACR (mg/g)	—	12.61 ± 5.8	1,160.07 ± 1,883.8	—	12.66 ± 7.7	69.81 ± 56.9	2,756.76 ± 2,087.4
24-hour urinary protein quantity (mg/24h)	—	37.92 ± 26.8	1,437.67 ± 2,298.2	—	48.36 ± 67.1	138.89 ± 295.2	3,555.62 ± 3,506.2

### Inclusion and Exclusion Criteria

Inclusion criteria include (1) 20-75 years old (2), All patients met the diagnostic criteria of 2-DM (3), The patients with microalbuminuria and macroalbuminuria met the diagnostic criteria of DN (4), eGFR >=90ml/min/1.73m2 in the 2-DM group, eGFR should be above 30ml/min/1.73m^2^ in both microalbuminuria group and macroalbuminuria group (5), Blood pressure below 140/90 mmHg (6), sign the informed consent.

Exclusion criteria include (1) Primary kidney disease with a definite diagnosis (2), Other systemic diseases that can cause proteinuria (3), Acute complications of diabetes mellitus and urinary tract infection in the past 1 month (4), Complicated with serious primary diseases in cardiovascular, cerebrovascular, liver, kidney, and the hematopoietic system as well as the tumor (5), Suffering from mental illness and unable to cooperate (6), Pregnant or lactating women, or those preparing for pregnancy (7), Women in their menstrual period (8), Those who have participated in other clinical trials within the past 1 month.

### Sample Preparation and Analysis

Serum samples were first thawed on ice. Briefly, 40 µL of serum was mixed with 225 µL of ice-cold MeOH. Each sample was then vortexed for 10 seconds and added to 750 µL of cold MTBE, and the mixtures were vortexed for 10 seconds before being shaken for 10 min at 4°C in an orbital mixer. After adding 188 µL of room-temperature LC/MS grade water, the samples were vortexed for 20 seconds and then centrifuged at 14,000 rcf at 4°C for 2 min. The upper liquid was transferred to fresh tubes and then dried in a SpeedVac sample concentrator at 45°C for 2 h. The dried lipids were redissolved in 100 µL of isopropyl alcohol/acetonitrile/water (30:65:5, *v/v/v*) mixture, and the samples were vortexed for 10 seconds and then centrifuged at 14,000 rcf at 4°C for 10 min. The mixture was then transferred to a sample vial with a glass insert and subjected to LC-MS analysis. Quality control (QC) samples were prepared by pooling equal amounts of lipid extracts from every sample, divided into aliquots, and analyzed every fifteen samples.

### Chromatography and MS

The analysis was performed on a UHPLC system (Shimazu Nexera X2 LC-30AD, Japan) coupled with an ESI-triple quadrupole mass spectrometer (SCIEX Triple Quad 5500+, Singapore).

Lipid separation was carried out using a Waters ACQUITY UPLC BEH HILIC (100 mm×2.1 mm I.D., 1.7 μm; Waters, Milford, MA, USA) column at 35 ° C with a flow rate of 500 µL/min, and the injection volume of each sample was 5 µL.

The mobile phase consisted of two solvents: 10 mM ammonium acetate (NH_4_OAc) in water: acetonitrile (5:95, *v/v*, pH adjustment usually not needed, A) and 10 mM ammonium acetate (NH_4_OAc) in water: acetonitrile (50:50, *v/v*, adjusted pH 8.2 with ammonium hydroxide, B). The lipids were separated with an optimized gradient elution: 0–10.0 min, 0.1%–20% B; 10.0–11.0 min, 20%–98% B; 11.0–13.0 min, 98% B; 13.0–13.1 min, 98%–0.1% B; 13.1–16.0 min, 0.1% B.

The mass spectrometer was operated under positive and negative switching ionization mode with an electrospray voltage (capillary voltage) of 4500/-4500 V. The MRM/retention time pairs were provided to the Scheduled MRM™ Algorithm to build the final MRM acquisition methods, and each MRM transition was monitored only during a short retention time window of 180 s. The typical source conditions were cohort: curtain gas as 35 and ion source temperature as 500 ° C. Ion source gas 1 (GS 1) and ion source gas 2 (GS 2) were all set at 50 and 60. The declustering potential was cohort at 80/-80 V. The collision cell exit potential was cohort at 9/-11 V in the positive or negative modes.

### Data Analysis

Raw data were acquired from Analyst^®^ 1.7.1 software (SCIEX) and then quantified with MultiQuant™ software. After removing the missing values using the 80% rule, the other missing values were replaced by 1/5 of each variable’s minimum positive value. Furthermore, all statistical analyses were carried out on log-transformed data, which were median normalized and Pareto scaled before the multivariate analysis. All steps were completed by MetaboAnalyst 5.0 (https://www.metaboanalyst.ca/). The identified lipids were further analyzed using univariate and multivariate statistical methods. The normalized data were imported into SIMCA software (version 14.1; Umetrics) and MetaboAnalyst 5.0 for partial least squares-discriminant analysis (PLS-DA) and orthogonal partial least squares-discriminant analysis (OPLS-DA), respectively. The significantly different lipid metabolites were identified based on variable importance in the projection (VIP) obtained from the OPLS-DA model and Student’s t-test (*p value*) with Benjamini-Hochberg-based false discovery rate (FDR). When the lipids met the criteria of VIP > 1.0, *p value* < 0.05 and FDR < 0.05 were considered differential metabolites.

Candidate metabolites were analyzed to identify potential diagnostic biomarkers. The forward stepwise binary logistic regression method and the Wald test were used to build the model based on the potential biomarkers. The diagnostic efficacy of the regression analysis results was analyzed and quantified by receiver operating characteristic (ROC) curve analysis. The area under the ROC curve (AUC) was calculated. Stepwise binary logistic regression and ROC curve analysis were performed with SPSS 25.0 software (SPSS, Inc.). GraphPad Prism 8 (GraphPad Software, La Jolla, CA, USA) was used to visualize individual metabolite levels in violin graphs.

## Results

**Figure 1:**
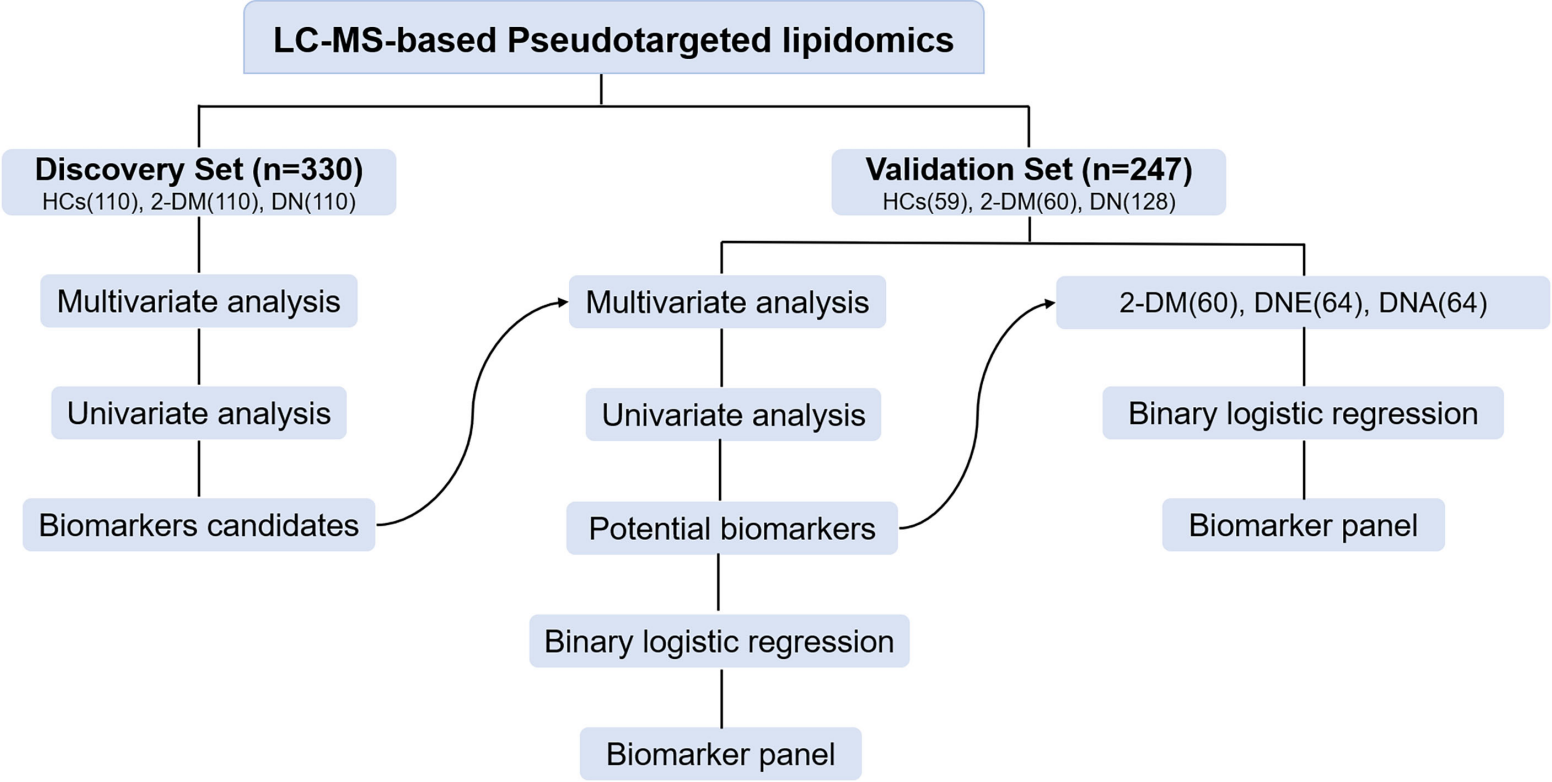
Design of the study.

In this study, a total of 330 serum samples were collected as a discovery cohort to find candidate biomarkers. Meanwhile, a total of 247 participants, including 59 HCs, 60 patients with 2-DM, and 128 patients with DN, including 64 patients with DNE and 64 patients with DNA, were enrolled as a validation cohort to test the identified biomarkers (**Figure 1**). The demographic characteristics and clinical information of the subjects are shown in **Table 1**.

### Serum Lipid Profiling of LC–MS

**Figure 2:**
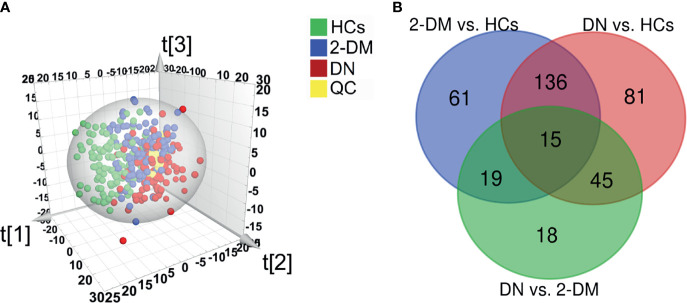
Identification of potential metabolic biomarkers for the diagnosis of DN. **(A)** Partial least squares-discriminant analysis (PLS-DA) score plot based on HCs (green), 2-DM (blue), DN (red) groups, and QC samples (yellow) in the Discovery Set. **(B)** Venn diagram displays the differential metabolites when the 2-DM and DN groups were compared with the HCs, and the DN groups was compared with the 2-DM in the Discovery Set.

In the initial pseudotargeted lipid metabolomics analysis, we examined 330 serum samples. In the metabolic spectrum, 1221 metabolites were identified, covering more than 21 subclasses. We further applied PLS-DA (**Figure 2A**) and OPLS-DA (**Supplementary Figure S1**) to identify the metabolic profile differences between groups in the discovery data cohort. All of the QC samples clustered closely, verifying the reliability of the present study. Without overfitting of the model (**Supplementary Figure S2**), the apparent separation among the HCs, 2-DM, and DN groups, cumulative R2Y at 0.641 and Q2 at 0.359, indicated that the lipid metabolism pattern was changed among the three groups. Based on the significant changes in the comparison among the lipid metabolites of HCs, 2-DM, and DN, multivariate and univariate statistical significance criteria (VIP >1, *p value* < 0.05, and FDR< 0.05) were applied to determine 231 metabolites of 2-DM *vs.* HCs, 277 metabolites of DN *vs.* HCs, and 97 metabolites of DN *vs.* 2-DM. Among them, there were 15 differential metabolites in the three comparisons (**Figure 2B**).

### Defining and Verifying Potential Biomarkers for DN

**Figure 3:**
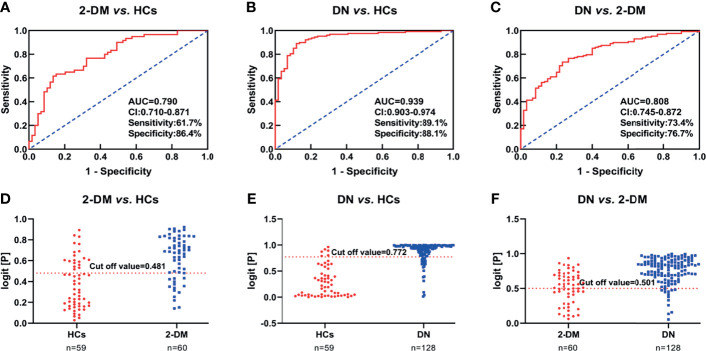
**(A–C)** Receiver operating characteristic curve analysis (ROC) in combination with LPE(16:0) and TAG54:2-FA18:1 to discriminate HCs, 2-DM and DN patients in the Validation Set. **(D–F)** Prediction accuracies of the panel of biomarkers (LPE(16:0) and TAG54:2-FA18:1) in the Validation Set. The area under the curve (AUC) is given at 95 % confidence intervals. AUC, area under the curve; CI, confidence interval.

We then further examined the above metabolites in the validation cohort to identify potential biomarkers and test their validity. There were 47 metabolites (**Supplementary Table S1**) with significant differences in the three comparisons (2-DM *vs.* HCs, DN *vs.* HCs, and DN *vs.* 2-DM). Eight of these metabolites showed expression trends consistent with our findings in the discovery cohort, including LPE(16:0), LPE(18:0), LPE(20:1), PE(16:0/18:1), PE(16:0/18:2), PE(16:0/20:2), TAG54:2-FA18:1, and TAG54:3-FA18:0. Details of these metabolites are listed in **Table 2**. Subsequently, using the eight potential biomarkers, binary logistic regression analysis with a forwarding stepwise optimization algorithm (Wald) was used to construct the optimal model. Finally, the combination of LPE(16:0) and TAG54:2-FA18:1 was selected as the ideal biomarker panel to distinguish HCs, 2-DM, and DN. The ideal biomarker panel showed sensitivity at 61.7% and 89.1%, specificity at 86.4% and 88.1%, and AUC at 0.790 and 0.939, respectively, to differentiate patients with 2-DM and DN from HCs (**Figures 3A, B**). The ideal biomarker panel showed a sensitivity of 73.4%, specificity of 76.7%, and AUC of 0.808 to differentiate 2-DM and DN (**Figure 3C**). The predictive value was 75.0% for 2-DM *vs.* HCs in the validation cohort (**Figure 3D**), 81.2% for DN *vs.* HCs in the validation cohort (**Figure 3E**), and 90.6% for DN *vs.* 2-DM in the validation cohort (**Figure 3F**).

**Table 2:** Identified differential metabolites between the 2-DM, DNE, DNA and health controls.

Metabolite	2-DM *vs.* HCs				DN *vs.* HCs				DN *vs.* 2-DM			
	VIP	*p value*	FDR	FC	VIP	*p value*	FDR	FC	VIP	*p value*	FDR	FC
LPE(16:0)	1.397	0.003	0.011	1.580	2.364	<0.001	<0.001	6.825	2.665	<0.001	<0.001	4.320
LPE(18:0)	1.361	0.007	0.022	2.006	2.231	<0.001	<0.001	5.072	2.025	<0.001	<0.001	2.528
LPE(20:1)	1.511	<0.001	0.002	2.927	2.126	<0.001	<0.001	5.707	1.859	<0.001	0.003	1.950
PE(16:0/18:1)	2.315	<0.001	<0.001	9.994	2.683	<0.001	<0.001	28.153	2.830	<0.001	<0.001	2.817
PE(16:0/18:2)	2.146	<0.001	<0.001	7.734	2.506	<0.001	<0.001	18.622	2.645	<0.001	0.001	2.408
PE(16:0/20:2)	2.347	<0.001	<0.001	9.186	2.693	<0.001	<0.001	28.255	2.412	<0.001	<0.001	3.076
TAG54:2-FA18:1	1.903	<0.001	<0.001	3.437	2.267	<0.001	<0.001	7.493	1.450	<0.001	0.002	2.180
TAG54:3-FA18:0	1.821	<0.001	<0.001	2.666	2.327	<0.001	<0.001	4.425	1.102	0.001	0.019	1.660

VIP, variable importance in the projection; FC, fold change; FDR, false discovery rate.

### Biomarkers for the Differential Diagnosis of DNE and DNA

**Figure 4:**
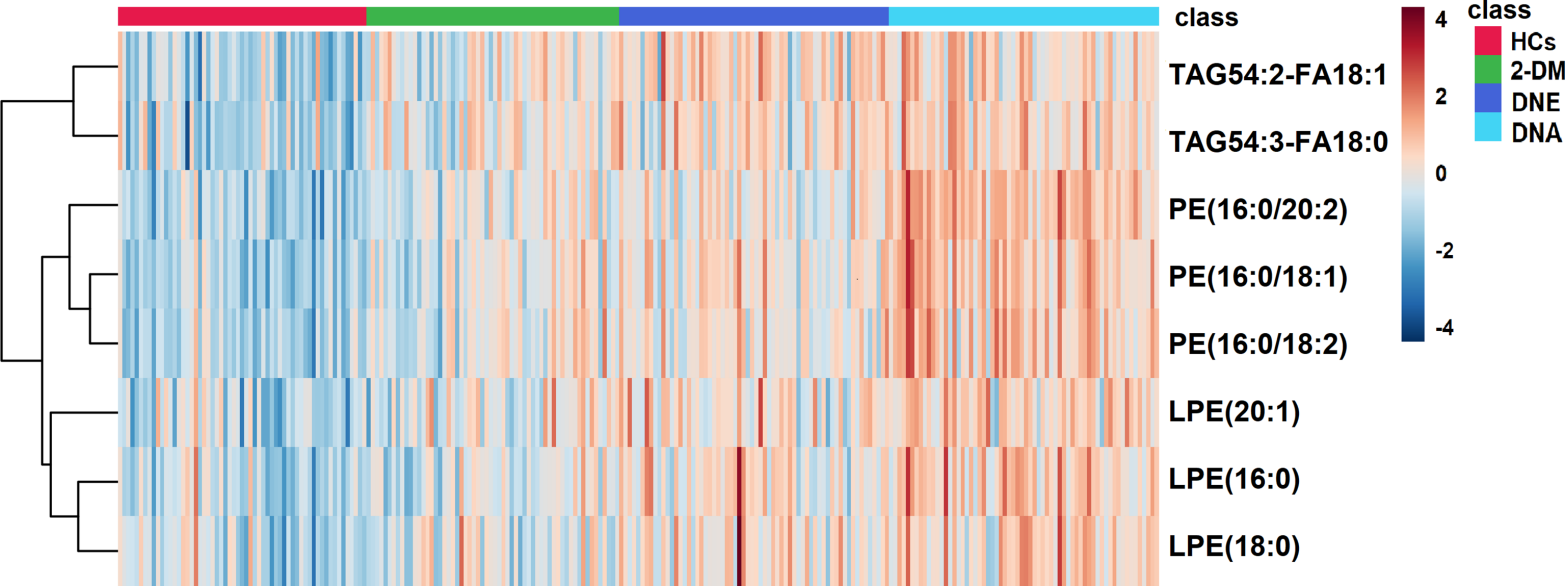
A Heatmap of the differential metabolites in HCs, 2-DM, DNE and DNA. Rows: serum samples; Columns: lipid species.

**Figure 5:**
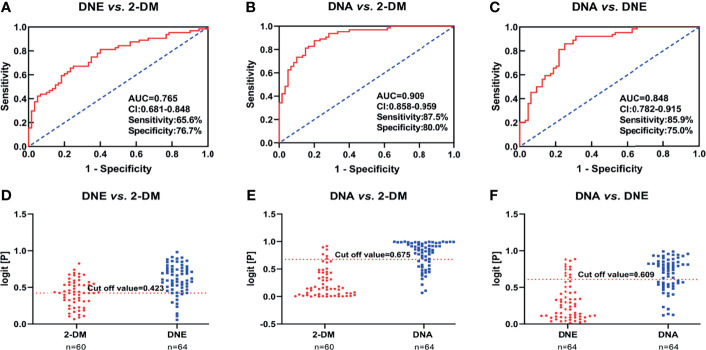
**(A–C)** Receiver operating characteristic curve analysis (ROC) in combination with LPE(16:0) and TAG54:2-FA18:1 to discriminate HCs, 2-DM and DN patients in the Validation Set. **(D–F)** Prediction accuracies of the panel of biomarkers (LPE(16:0) and TAG54:2-FA18:1) in the Validation Set. The area under the curve (AUC) is given at 95 % confidence intervals. AUC, area under the curve; CI, confidence interval.

**Figure 6:**
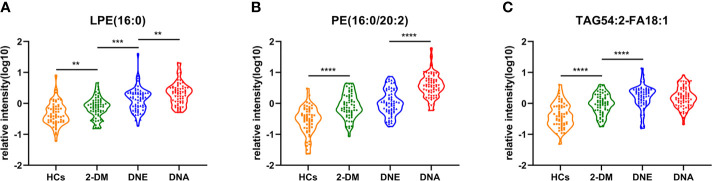
Serum relative intensity of LPE(16:0) **(A)**, PE(16:0/20:2) **(B)**, and TAG54:2-FA18:1 **(C)** in the HCs (orange), 2-DM (green), DNE (blue) and DNA (red). **P < 0.01, ***P < 0.001, and ****P < 0.0001.

We further divided participants with DN in the validation cohort into DNE and DNA to determine if there were ideal biomarkers among these potential biomarkers that could distinguish 2-DM, DNE, and DNA. First, a heat map was used to find the relative intensity distribution of the eight potential biomarkers in HCs, 2-DM, DNE, and DNA, as shown in **Figure 4**. The serum levels of these metabolites in HCs, 2-DM, DNE, and DNA increased with the severity of the disease. On this basis, eight potential biomarkers were used to perform binary logistic regression analysis using a forward stepwise optimization algorithm (Wald) for the construction of optimal models for DNE *vs.* 2-DM, DNA *vs.* 2-DM, and DNA *vs.* DNE. The results showed that the combination of LPE(16:0), PE(16:0/20:2), and TAG54:2-FA18:1 could distinguish 2-DM, DNE, and DNA very well. The ideal biomarker panel showed a sensitivity of 65.6%, specificity of 76.7%, and AUC of 0.765 to differentiate 2-DM and DNE (**Figure 5A**). Similarly, between 2-DM and DNA, we showed a sensitivity of 87.5%, specificity of 80.0%, and AUC of 0.909 (**Figure 5B**); between DNE and DNA, the sensitivity index was 85.9%, the specificity index was 75.0%, and the AUC index was 0.848 (**Figure 5C**). Predictive values of 82.8%, 70.3%, and 64.1% were found for DNE *vs.* 2-DM, DNA *vs.* 2-DM, and DNA *vs.* DNE in the validation cohort by setting 0.423, 0.675, and 0.609 as the optimal cutoff values (**Figures 5D** **–F**). LPE(16:0), PE(16:0/20:2), and TAG54:2-FA18:1 levels were gradually increased in the candidates from HCs, 2-DM, DNE, and DNA (**Figure 6**). To further validate candidates that might be useful in detecting DN, we analyzed the relationship between each lipid species and eGFR, Scr, and UAE. The analysis showed that LPE(16:0) and PE(16:0/20:2) were negatively correlated with eGFR (r=-0.2161, P<0.001; r=-0.5206, P<0.001). LPE(16:0) and PE(16:0/20:2) were positively correlated with Scr (r=0.1613, P=0.013; r=0.3816, P<0.001). PE(16:0/20:2) was positively correlated with UAE (r=0.3028, P<0.001). In addition, the association analysis between UAE, Scr or eGFR, and lipidomes showed no significant correlation.

## Discussion

DN is a diabetic complication characterized by progressive kidney damage. Clinical treatment requires multimedication, and kidney replacement therapy imposes enormous economic burdens on the health care system (27). In this field, it is well known that DN patients have a higher mortality rate than DM patients without kidney damage (28). Therefore, early diagnosis and intervention to slow down the progression of DN will be of great significance to reduce the occurrence of unpredictable vascular events and to improve the survival rate and quality of life. DN is usually diagnosed as increased UACR and/or decreased eGFR, excluding primary and secondary CKD. Renal biopsy is the most accurate method for diagnosing DN, but in clinical practice, renal biopsy in DM patients is still rare because of its invasiveness (29). Since the accuracy and specificity of the current diagnostic criteria for DN cannot meet our requirements, an ideal diagnostic marker for DN, especially for the early stage of DN, is urgently needed. In this study, we performed a comprehensive study of lipids in the serum of HCs and 2-DM, DNE, and DNA individuals using pseudotargeted lipid metabolomics. A total of 1221 serum lipid metabolites were identified.

We then tested the lipid metabolites related to the occurrence and development of DN in the validation cohort. Compared with HCs and 2-DM patients, significantly increased levels of LPE(16:0), LPE(18:0), LPE(20:1), PE(16:0/18:1), PE(16:0/18:2), PE(16:0/20:2), TAG54:2-FA18:1, and TAG54:3-FA18:0 were observed in DN patients. Patients with CKD have previously been reported to exhibit disorders of glycerolipid metabolism and glycerophospholipid metabolism (30, 31).

PE(16:0/20:2) is a phosphatidylethanolamine(PE), which combinations of one chain of palmitic acid and one chain of eicosadienoic acid attached at the C-1 and C-2 positions, respectively. PE is the second most abundant and multifunctional glycerophospholipid in eukaryotic cells (32). It is essential in mammalian development and cellular processes, including being involved in metabolism and signaling (33). PE and cholesterol can improve the hardness of the bilayer membrane, which indicates that PE and cholesterol could maintain the fluidity of the cell membrane. Phosphatidylethanolamine n-methyltransferase (PEMT) is a crucial enzyme that promotes PC synthesis and PE conversion to PC. Once the PC: PE ratio is decreased, ER stress and SREBP1 are activated. ER stress is associated with insulin resistance (IR) and 2-DM (34, 35). Furthermore, once PE undergoes glycosylation due to the presence of free amine groups, it may increase the oxidation sensitivity in the case of hyperglycemic conditions (36). Additionally, to promote lipid peroxidation, glycated PE partially produces ROS, which is associated with inflammation and other DM complications, such as DN (37, 38).

When the PE: PC (phosphatidylcholine) ratio increases, the fluidity of the cell membrane decreases significantly. As a consequence, the increase in permeability of the cell membrane causes cell damage (39). This imbalance of the membrane lipid composition affects the characteristics of the membrane and induces pathological changes in erythrocyte membranes in patients with 2-DM (40).

Lysophosphatidylethanolamine (LPE) is a lysophospholipid product of partial hydrolysis of PE catalyzed by phospholipase A2 (PLA2) in glycerophospholipid metabolism (41). LPE(16:0) as an LPE, is mainly involved in the Phospholipid Biosynthesis. Investigation of existing literature, alteration of LPE (16:0) also was found in iron deficiency, ulcerative colitis, and colorectal cancer, but the specific mechanism of action remains unclear (42, 43). Before this, no such differences in the metabolism of LPE(16:0) have been reported in DM and DN. We speculated that LPE (16:0) might play a role in renal damage through its metabolites, basis the following information. LPE is converted to lysophosphatidic acid (LPA) by the action of lysophospholipase D (Lyso PLD). LPA can activate endothelial cells and initiate the secretion of a variety of proinflammatory peptides and proteins, in addition to causing the rupture of red blood cells and other cells, leading to hemolysis, cell necrosis, and organ damage, such as kidney disease (44). It has been reported in the literature that the LPA-LPAR axis mainly induces pathological changes in the structure and function of renal cells (45).

Consistent with previous studies, the TAG level was elevated in patients with 2-DM and CKD compared to healthy subjects (46, 47). TAG biosynthesis occurs *via* the glycerolipid metabolic pathway of fatty acids (FAs) to produce LPA, which is further transformed into phosphatidic acid (PA). PA is then hydrolyzed to form diacylglycerols (DAGs) and finally esterified to TAGs (48, 49). It has been reported that TAG and DAG may contribute to insulin resistance by a similar mechanism as the stimulation of β-cell apoptosis by free fatty acids (FFAs) *via* c-Jun N-terminal kinase (JNK) (50). KEGG reactions in human pathways involving TAG54:2-FA18:1, Phospholipid + 1,2-Diacyl-sn-glycerol <=> Lysophospholipid + Triacylglycerol, verify the interconnection between PE, LPE, and TAG, and whether these metabolic changes broke the balance of this reaction, and then triggered a series of metabolic diseases. Unfortunately, the specific mechanism of which needs further research.

This lipid metabolomics provides a strategy for DN diagnosis in the clinic. The results can be used as a reference for further clinical examination. However, this study does have its limitations. First, all participants were Asian and enrolled from the same center, and because both 2-DM and DN were accompanied by obesity, resulting in significant differences between groups in terms of IBM and age, which may limit the applicability of our conclusions. Second, lipidomics analysis has limitations, and the results need to be further verified in additional studies. In future studies, the patients should be expanded to include other races and ethnicities across multiple research centers. The number of participants should be increased and information on their renal function parameters should be followed up to make the results more compelling.

In summary, we found that lipid metabolism disorders in DN were associated with LPE, PE, and TAG changes. A biomarker panel comprised of LPE(16:0), PE(16:0/20:2), and TAG54:2-FA18:1 was identified and further validated by a longitudinal sectional study for the diagnosis of DN, which showed that LPE(16:0), PE(16:0/20:2), and TAG54:2-FA18:1 were positively correlated with the severity of the development of DN. This biomarker panel can identify DN patients and distinguish DNA and DNE patients from HCs and 2-DM individuals. Therefore, it is proposed that this lipid biomarker panel has great potential in the diagnosis and treatment of DN in the clinical setting.

## Data Availability Statement

The original contributions presented in the study are included in the article/**Supplementary Material**. Further inquiries can be directed to the corresponding author.

## Ethics Statement

The studies involving human participants were reviewed and approved by Institutional Review Board and the Ethics Committee of the First Affiliated Hospital of Nanjing University of Traditional Chinese Medicine. The patients/participants provided their written informed consent to participate in this study.

## Author Contributions

TX, Conceptualization, Formal analysis, and Writing - Original Draft Preparation. XX and LuZ, Methodology, Data curation, and Writing - Review & Editing. KZ, QW, YY, and LiZ, Formal analysis and Validation. LL, LX, WQ, JW, and MK Investigation and Resources. XA, Funding acquisition; SL, Conceptualization, Project administration, and Funding acquisition. All authors contributed to the article and approved the submitted version.

## Funding

This work is financially supported by the National Natural Science Foundation of China (No. 81774248 and No.82074359). The Open Projects of the Discipline of Chinese Medicine of Nanjing University of Chinese Medicine Supported by the Subject of Academic Priority Discipline of Jiangsu Higher Education Institutions (No. ZYX03KF031 and No. ZYX03KF027).

## Conflict of Interest

The authors declare that the research was conducted in the absence of any commercial or financial relationships that could be construed as a potential conflict of interest.

## Publisher’s Note

All claims expressed in this article are solely those of the authors and do not necessarily represent those of their affiliated organizations, or those of the publisher, the editors and the reviewers. Any product that may be evaluated in this article, or claim that may be made by its manufacturer, is not guaranteed or endorsed by the publisher.
