# Supplementary material for: Lipidomics Reveals Serum Specific Lipid Alterations in Diabetic Nephropathy
Source: Front Endocrinol (Lausanne). 2021 Dec 9;12:781417. doi: 10.3389/fendo.2021.781417 (PMC8695735; doi:10.3389/fendo.2021.781417)
Supplement: Supplementary file 1 [file DataSheet_1.docx]

**SUPPLEMENTARY DATA**

**Table S1.** Differential metabolites between HCs, 2-DM, and DN in the Validation Set.

| Metabolite | VIP | | | FDR | | | FC | | |
| --- | --- | --- | --- | --- | --- | --- | --- | --- | --- |
|  | 2-DM *vs.* HCs | DN *vs.* HCs | DN *vs.* 2-DM | 2-DM *vs.* HCs | DN *vs.* HCs | DN *vs.* 2-DM | 2-DM *vs.* HCs | DN *vs.* HCs | DN *vs.* 2-DM |
| LPE(16:0) | 1.397 | 2.364 | 2.665 | 0.011 | <0.001 | <0.001 | 1.580 | 6.825 | 4.320 |
| LPE(18:0) | 1.361 | 2.231 | 2.025 | 0.022 | <0.001 | <0.001 | 2.006 | 5.072 | 2.528 |
| LPE(18:1) | 1.874 | 2.446 | 2.530 | <0.001 | <0.001 | <0.001 | 4.439 | 10.587 | 2.385 |
| LPE(20:1) | 1.511 | 2.126 | 1.859 | 0.002 | <0.001 | 0.003 | 2.927 | 5.707 | 1.950 |
| LPE(20:2) | 1.581 | 1.999 | 2.142 | 0.001 | <0.001 | 0.005 | 3.704 | 7.187 | 1.941 |
| PE(16:0/16:0) | 1.586 | 2.359 | 2.502 | 0.003 | <0.001 | <0.001 | 3.197 | 10.179 | 3.184 |
| PE(16:0/18:1) | 2.315 | 2.683 | 2.830 | <0.001 | <0.001 | <0.001 | 9.994 | 28.153 | 2.817 |
| PE(16:0/18:2) | 2.146 | 2.506 | 2.645 | <0.001 | <0.001 | 0.001 | 7.734 | 18.622 | 2.408 |
| PE(16:0/18:3) | 1.474 | 2.319 | 2.300 | 0.003 | <0.001 | <0.001 | 4.038 | 9.998 | 2.476 |
| PE(16:0/20:1) | 2.059 | 2.682 | 2.420 | <0.001 | <0.001 | <0.001 | 3.611 | 11.347 | 3.143 |
| PE(16:0/20:2) | 2.347 | 2.693 | 2.412 | <0.001 | <0.001 | <0.001 | 9.186 | 28.255 | 3.076 |
| PE(16:0/20:3) | 2.046 | 2.467 | 2.553 | <0.001 | <0.001 | <0.001 | 5.174 | 14.560 | 2.814 |
| PE(16:0/20:4) | 2.084 | 2.513 | 2.259 | <0.001 | <0.001 | 0.001 | 8.121 | 16.571 | 2.041 |
| PE(16:0/22:4) | 1.615 | 2.095 | 2.535 | 0.001 | <0.001 | 0.001 | 3.842 | 8.881 | 2.312 |
| PE(16:0/22:5) | 1.763 | 2.394 | 2.542 | <0.001 | <0.001 | <0.001 | 4.283 | 11.595 | 2.707 |
| PE(16:0/22:6) | 1.801 | 2.256 | 1.494 | <0.001 | <0.001 | 0.026 | 3.601 | 6.440 | 1.788 |
| PE(18:0/16:0) | 1.944 | 2.424 | 2.602 | <0.001 | <0.001 | <0.001 | 4.417 | 14.464 | 3.274 |
| PE(18:0/18:1) | 2.483 | 2.798 | 2.394 | <0.001 | <0.001 | <0.001 | 9.727 | 26.749 | 2.750 |
| PE(18:0/18:2) | 2.335 | 2.678 | 2.415 | <0.001 | <0.001 | 0.001 | 13.356 | 28.930 | 2.166 |
| PE(18:0/18:3) | 1.612 | 2.188 | 1.760 | <0.001 | <0.001 | 0.003 | 2.786 | 5.649 | 2.027 |
| PE(18:0/20:1) | 2.067 | 2.500 | 1.845 | <0.001 | <0.001 | 0.003 | 4.917 | 11.162 | 2.270 |
| PE(18:0/20:2) | 2.113 | 2.533 | 2.044 | <0.001 | <0.001 | <0.001 | 7.608 | 19.228 | 2.528 |
| PE(18:0/20:3) | 1.894 | 2.387 | 2.184 | <0.001 | <0.001 | <0.001 | 5.158 | 11.486 | 2.227 |
| PE(18:0/20:4) | 2.326 | 2.667 | 1.998 | <0.001 | <0.001 | 0.001 | 9.164 | 20.607 | 2.249 |
| PE(18:0/22:4) | 1.816 | 2.376 | 2.232 | <0.001 | <0.001 | <0.001 | 3.260 | 8.165 | 2.504 |
| PE(18:0/22:5) | 1.994 | 2.379 | 1.941 | <0.001 | <0.001 | 0.002 | 6.922 | 15.117 | 2.184 |
| PE(18:1/18:1) | 2.163 | 2.716 | 2.696 | <0.001 | <0.001 | <0.001 | 6.771 | 20.727 | 3.061 |
| PE(18:1/18:2) | 2.282 | 2.697 | 2.619 | <0.001 | <0.001 | <0.001 | 10.323 | 25.938 | 2.513 |
| PE(18:1/18:3) | 1.770 | 2.341 | 1.989 | <0.001 | <0.001 | 0.001 | 7.478 | 15.233 | 2.037 |
| PE(18:1/20:1) | 2.387 | 2.690 | 2.100 | <0.001 | <0.001 | 0.002 | 9.736 | 20.004 | 2.055 |
| PE(18:1/20:2) | 2.075 | 2.665 | 2.320 | <0.001 | <0.001 | 0.001 | 8.435 | 17.727 | 2.102 |
| PE(18:1/20:3) | 1.716 | 2.334 | 2.435 | <0.001 | <0.001 | 0.001 | 6.077 | 12.426 | 2.045 |
| PE(18:1/20:4) | 2.225 | 2.600 | 2.202 | <0.001 | <0.001 | 0.004 | 11.127 | 21.915 | 1.970 |
| PE(18:1/22:4) | 1.175 | 2.081 | 2.373 | 0.016 | <0.001 | <0.001 | 3.025 | 7.007 | 2.317 |
| PE(18:1/22:5) | 1.638 | 2.310 | 2.384 | 0.001 | <0.001 | <0.001 | 4.632 | 11.390 | 2.459 |
| PE(18:1/22:6) | 2.062 | 2.534 | 1.702 | <0.001 | <0.001 | 0.005 | 6.128 | 11.553 | 1.885 |
| PE(18:2/20:1) | 2.032 | 2.542 | 2.334 | <0.001 | <0.001 | 0.002 | 6.877 | 15.561 | 2.263 |
| PE(18:2/20:2) | 1.655 | 2.284 | 2.210 | <0.001 | <0.001 | 0.003 | 5.174 | 10.863 | 2.099 |
| PE(18:2/20:3) | 1.675 | 1.934 | 2.008 | 0.001 | <0.001 | 0.040 | 3.610 | 6.155 | 1.705 |
| PE(18:2/20:4) | 1.339 | 2.121 | 2.309 | 0.005 | <0.001 | 0.001 | 2.678 | 6.187 | 2.310 |
| TAG54:1-FA18:0 | 1.766 | 1.876 | 1.005 | <0.001 | <0.001 | 0.050 | 3.773 | 6.517 | 1.727 |
| TAG54:1-FA18:1 | 1.731 | 1.940 | 1.123 | <0.001 | <0.001 | 0.042 | 3.134 | 5.643 | 1.800 |
| TAG54:2-FA18:0 | 2.169 | 2.283 | 1.108 | <0.001 | <0.001 | 0.018 | 3.645 | 6.823 | 1.872 |
| TAG54:2-FA18:1 | 1.903 | 2.267 | 1.450 | <0.001 | <0.001 | 0.002 | 3.437 | 7.493 | 2.180 |
| TAG54:3-FA18:0 | 1.821 | 2.327 | 1.102 | <0.001 | <0.001 | 0.019 | 2.666 | 4.425 | 1.660 |
| TAG54:3-FA18:1 | 1.324 | 1.979 | 1.468 | 0.001 | <0.001 | 0.025 | 2.135 | 3.630 | 1.700 |
| TAG56:4-FA18:1 | 1.457 | 2.006 | 1.393 | <0.003 | <0.001 | 0.029 | 2.567 | 5.347 | 2.083 |

VIP: variable importance in the projection, FC: fold change, FDR: false discovery rate.


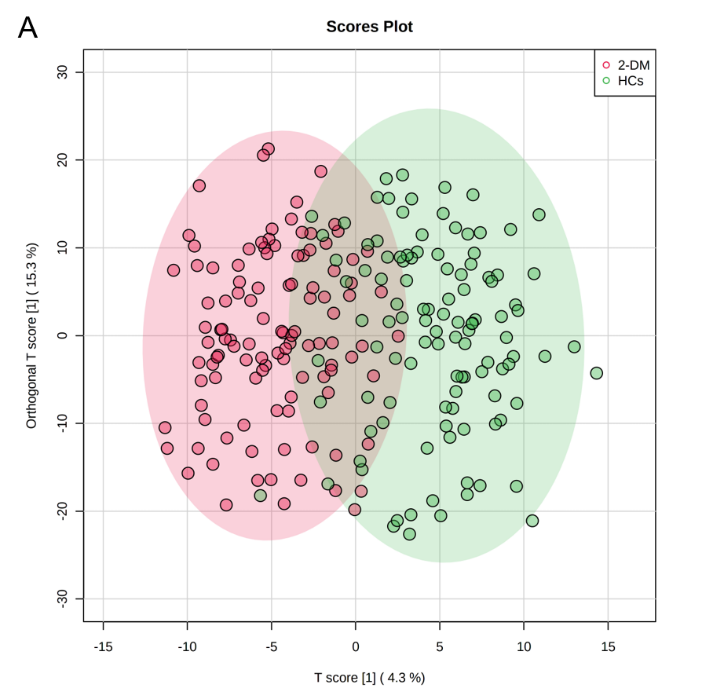

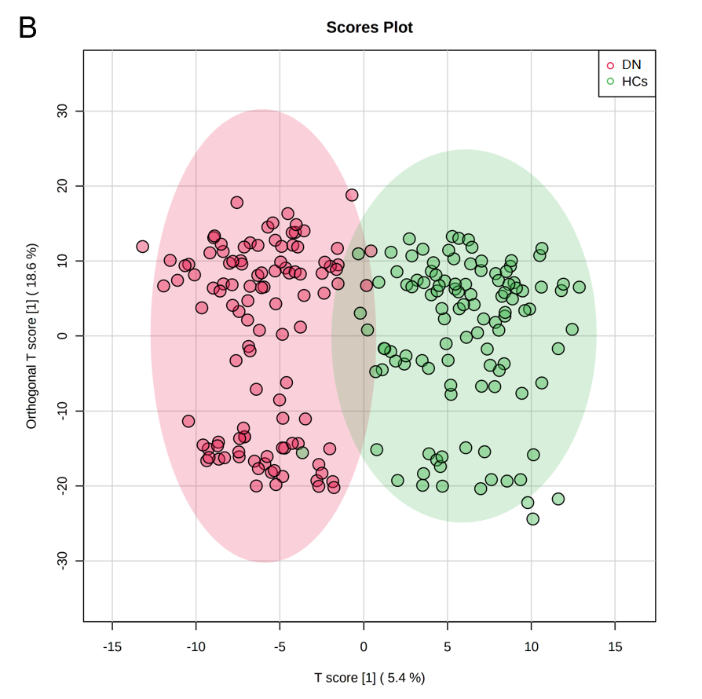


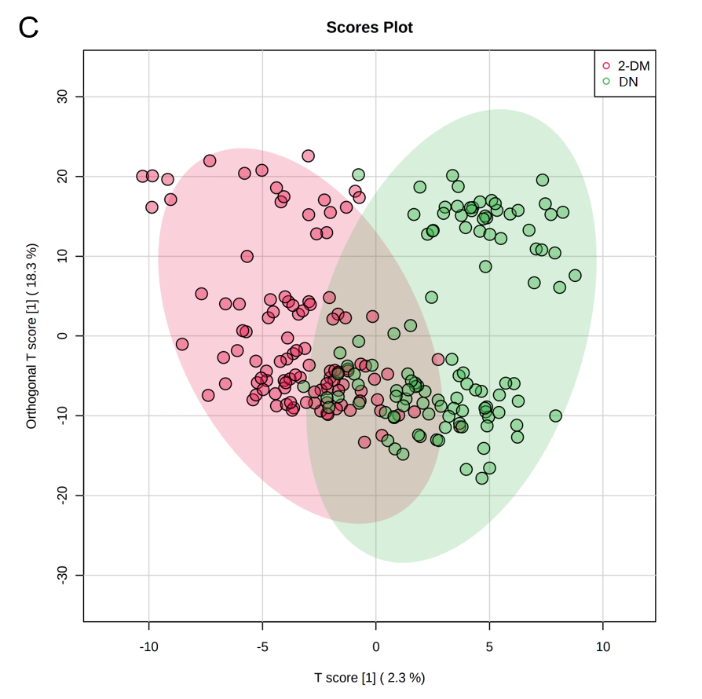


**Figure S1.** Orthogonal partial least squares discriminant analysis (OPLS-DA) score plot based on HCs, 2-DM, and DN groups in the Discovery Set.


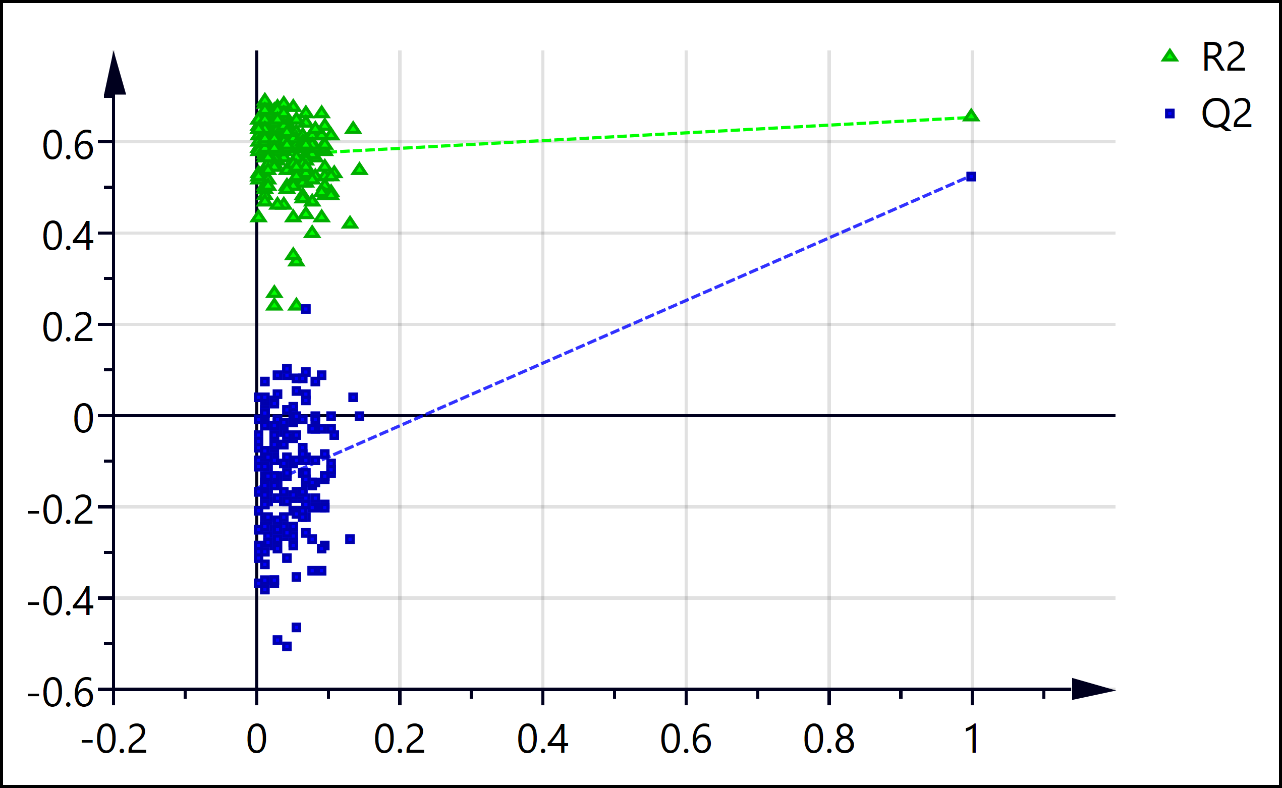


**Figure S2.** Cross-validation plot with a permutation test repeated 200 times. The intercepts of R2= (0.0, 0.569) and Q2= (0.0, - 0.16) illustrate the PLS-DA model is not over-fitting.
